# Evaluation of the pectoralis major flap for reconstructive head and neck surgery

**DOI:** 10.1186/1758-3284-3-12

**Published:** 2011-02-27

**Authors:** Astrid L Kruse, Heinz T Luebbers, Joachim A Obwegeser, Marius Bredell, Klaus W Grätz

**Affiliations:** 1 Department of Craniomaxillofacial and Oral Surgery, University of Zurich, Switzerland

## Abstract

**Purpose:**

The pectoralis major myocutaneous flap (PMMF) is a commonly used flap in reconstructive head and neck surgery, but in literature, the flap is also associated with a high incidence of complications in addition to its large bulk. The purpose of the study is the evaluation of the reliability and indication of this flap in reconstructive head and neck surgery.

**Patients and methods:**

The records of all patients treated with a PMMF between 1998 and 2009 were systematically reviewed. Data of recipient localization, main indication, and postoperative complications were analyzed.

**Results:**

The male to female ratio was 17:3, with a mean age of 60 years (45-85). Indications in 7 patients were recurrence of a squamous cell carcinoma, in one case an osteoradionecrosis and in 12 cases an untreated squamous cell carcinoma. In 6 male patients (30%), a complication appeared leading to another surgery.

**Conclusion:**

The PMMF is a flap for huge defects in head and neck reconstructive surgery, in particular when a bulky flap is needed in order to cover the carotid artery or reconstructive surgery, but the complication rate should not be underestimated in particular after radiotherapy.

## Introduction

The pectoralis major myocutaneous flap (PMMF) is a commonly used flap for reconstructive head and neck surgery. Ariyan was among the first to use this pedicle flap for head and neck defects [1,2]. Nowadays, free flaps are more common due to improved microsurgical techniques, but in several cases the PMMF still has its advantages, including its proximity to the head and neck, the simplicity of harvesting, and its use as an alternative when microsurgical flap failure occurs. The disadvantages can include a reduced neck mobility and the need to rotate the vascular pedicle of the flap 180° when using the skin paddle to resurface the neck. Another disadvantage can be the thickness of the flap, which is determined by the amount of subcutaneous fat between the pectoralis muscle and the overlying skin paddle, leading to possible reduced swallowing or speech function. On the other hand, in particular for cases like coverage of a reconstruction plate or coverage of the carotid artery, the bulkiness of PMMF can be an advantage. The PMMF is characterized by a simple procedure and a short time to harvest, but a simultaneous two-team approach is difficult in comparison to the classical forearm or anterolateral thigh flap.

Because of high complication rates in literature [3-13], the aim of the current study is to evaluate and compare the indications and the reliability for this flap in our department.

## Patients and methods

The records of all patients treated with a PMMF between 1998 and 2009 in the Clinic for Craniomaxillofacial and Oral Surgery, at the University Hospital in Zurich were systematically reviewed. The criterion for inclusion was performed PMMF, and for exclusion, inadequate information. Data concerning recipient localization, main indication, and postoperative complications were analyzed.

Major complications were evaluated if revision surgery was necessary and minor ones if conservative wound care alone was required.

### Surgical technique

First, the clavicle, xiphoid, ipsilateral sternal border are identified, and then the size and location of the skin paddle being located at the inferior-medial border of the pectoralis major muscle are marked. The vascular axis is drawn on the skin of the chest.

Second, the initial incision is made at the lateral part toward the anterior axillary line down to the pectoralis major muscle.

**Figure 1:**
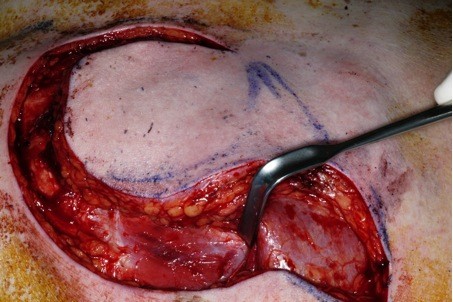
**Incision of the flap through the skin, subcutaneous fat and pectoralis fascia down to the chest wall**.

**Figure 2:**
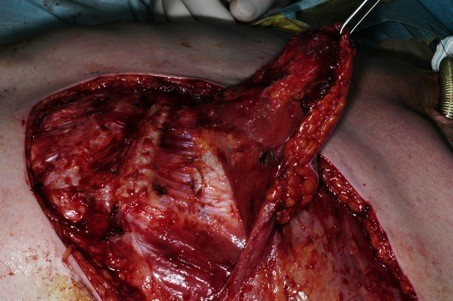
**Dissection of the flap off the chest wall**.

The maximum amount of muscle should be harvest, because the larger the muscle volume, the safer the flap due to the increased number of myocutaneous perforators (Figure 1). Third, the inferior, medial and lateral incisions are made through the skin, subcutaneous fat and pectoralis fascia down to the chest wall (Figure 2).

The superior incision is made down to the muscle fibres and the skin island is tightened to the muscle with absorbable sutures to protect the skin island during operative handling.

**Figure 3:**
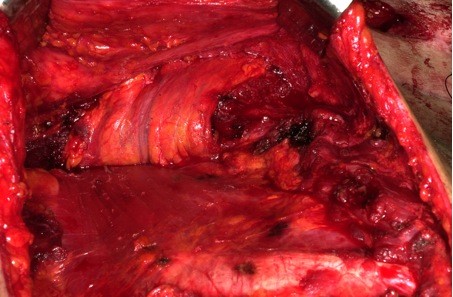
**Identification of the pedicel by visualization on the deep surface of the muscle**.

As the muscle is elevated inferiorly to superiorly, the pedicle should be identified by palpation and visualization on the deep surface of the muscle (Figure 3). The pectoralis major muscle derives its blood supply from the pectoral branch of the thoracoacromial artery and lateral thoracic artery. The thoracoacromial artery devides into four branches: pectoral, acromial, clavicular and deltoid. When the muscle fibres are cut along the sternal attachment, special attention should be taken not to cut the internal mammary perforators adjacent to the sternum that supply the deltopectoral flap. During the dissection the vascular bundle should always be seen in order to avoid injury to this bundle.

**Figure 4:**
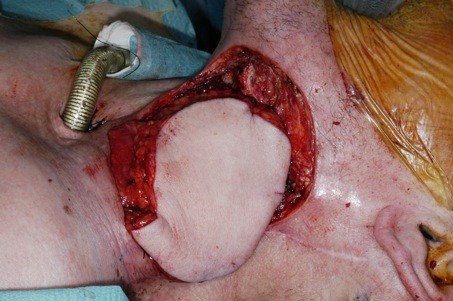
**Flap is being passed underneath the skin bridge**.

After dissection the flap off the chest wall, a subcutaneous tunnel is formed under the skin between neck (preserving the perforators to the overlying deltopectoral flap) and the chest and the flap is passed underneath the skin bridge (Figure 4).

Magrim et al. recommend in difficult cases, such as in patients with bulky flaps to use sterile liquid vaseline to lubricate the flap and to raise the ipsilateral shoulder in order to facilitate passage and during the procedure, to instill a vasodilator substance (papaverine or lidocaine) over the flap pedicle [14].

#### Variations

A myofascial flap can be raised without a skin paddle. In female patients the flap is below the breast.

In order to gain additional length, the skin paddle may be extended as a random-pattern flap beyond the inferior edge of the muscle belly or the clavicular portion of the pectoralis major muscle can be devided above the pedicle by debulking the muscle fibres over the proximal pedicle. Another alternative is to resect the middle third of the clavicle.

**Figure 5:**
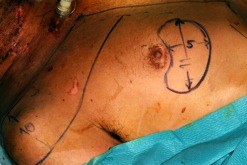
**Possibility of harvesting a deltopectoral and pectoralis major flap from the same side**.

In cases of a deltopectoral flap, this flap should be first harvested from its distal part, at least to the medial aspect of the thoracoacromial artery. It is possible to use both, deltopectoral and pectoralis major flap from the same side (Figure 5). The lateral thoracic artery should be preserved by dividing the humeral head of the pectoralis major muscle and the lateral border of the pectoralis minor muscle [15].

## Results

Between 1998 and 2009, 20 reconstructions utilizing PMMF were performed by four different surgeons. The patients' male to female ratio was 17:3, and the mean age was 60 years (45-85).

**Figure 6:**
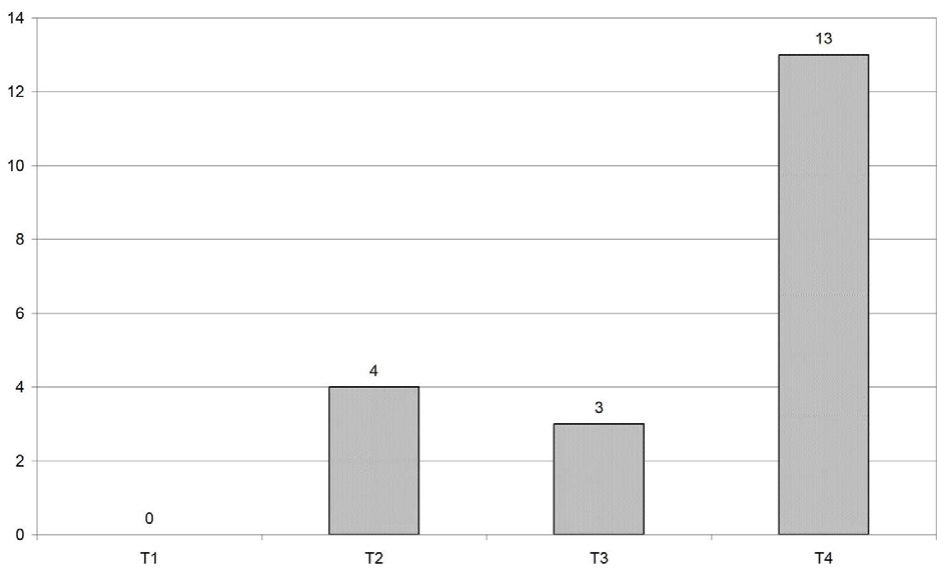
**Distribution of primary T status**.

Indications in 7 patients were a recurrence of a squamous cell carcinoma, in one case an osteoradionecrosis in order to cover exposed bone, and in 12 cases an untreated squamous cell carcinoma. The primary T status is listed in Figure 6. The main portion (13/19) was a T4 status.

**Figure 7:**
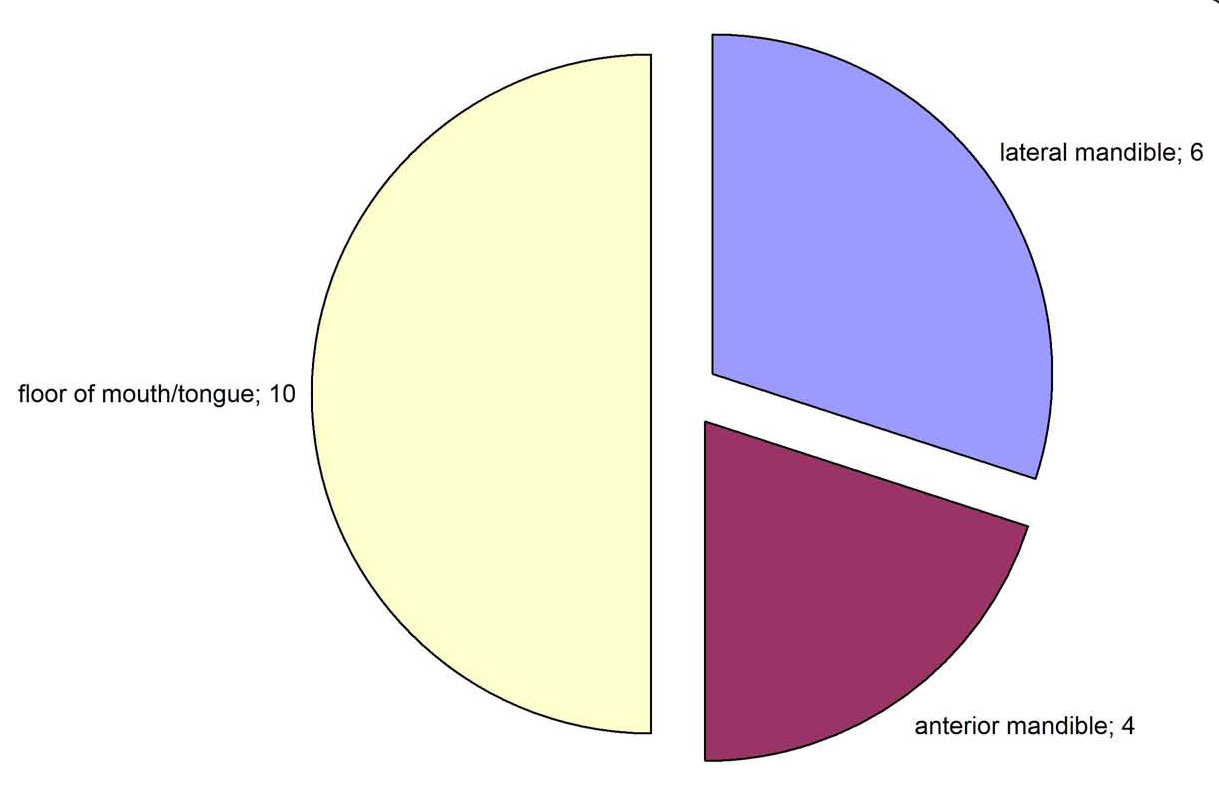
**Distribution of defect localizations covered with PMMF**.

The defect site distribution is shown in Figure 7. In this study mainly defects of the floor of the mouth or tongue were covered (50% of all sites).

In 6 male patients, a complication appeared, leading to another surgery (Table 1).

**Table 1:** Reported overall patient group

Gender	Age (years)	Indication	Localization	Radiotherapy	Complications
M	56	Recurrence	Mandible	Prior	Bleeding (minor)

M	54	Second oral cancer	Mandible	Prior, contralateral	Partial necrosis

M	64	Recurrence	Floor of mouth	Prior	-

M	48	Oral cancer	Floor of mouth	-	Necrosis, flap loss

M	51	Recurrence	Mandible	Prior	Complete necrosis

M	76	Recurrence	Mandible	Prior	Hematoma

M	56	Oral cancer	Floor of mouth	-	-

M	68	Recurrence	Mandible	Prior	-

M	45	Oral cancer	Chin	-	-

F	62	Recurrence	Mandible	-	-

M	55	Oral cancer	Floor of mouth	-	-

M	60	Osteomyelitis, Coverage of exposed bone	Mandible	Prior	Partial necrosis with infection

F	68	Oral cancer	Mandible	-	-

M	67	Oral cancer	Floor of mouth/tongue	-	-

M	58	Oral cancer	Floor of mouth	-	-

F	75	Oral cancer		-	-

M	53	Oral cancer	Floor of mouth	-	Hematoma

M	60	Oral cancer	Floor of mouth	-	-

M	61	Oral cancer	Floor of mouth	-	-

M	56	Recurrence	Floor of mouth	Prior	-

## Discussion

Several modifications have been suggested for multiple purposes. Some authors used only the pure muscle flap without skin, the pectoralis major myofascial flap, in order to reduce the thickness [16,17]. However concerning the bulkiness of the flap, a 50% reduction within 3 months is reported due to atrophy after division of the motor nerves [7].

Others included a segment from the fifth rib in the flap [18-20], but in cases of postoperative radiotherapy, this is not recommended [19]. Of course the flap can be combined with a non-vascularized bone graft, such as a free iliac crest brought out simultaneously [21]. In the current study, none of the patients had a bone graft inserted at the same time.

In females the use of an inframammary incision is recommended for aesthetic reasons [13]: but in the present study the PMMF was performed on only 3 female patients. Chaturvedi et al. described a technique whereby the flap was harvested through the skin paddle incision alone [22].

The double paddle modification as described by Freeman et al. [23] is sometimes an alternative to using another flap technique [24]. However, combinations of PMMF and radial forearm flap, fibula flap, and anterolateral thigh flap were successfully performed [25,26].

**Figure 8:**
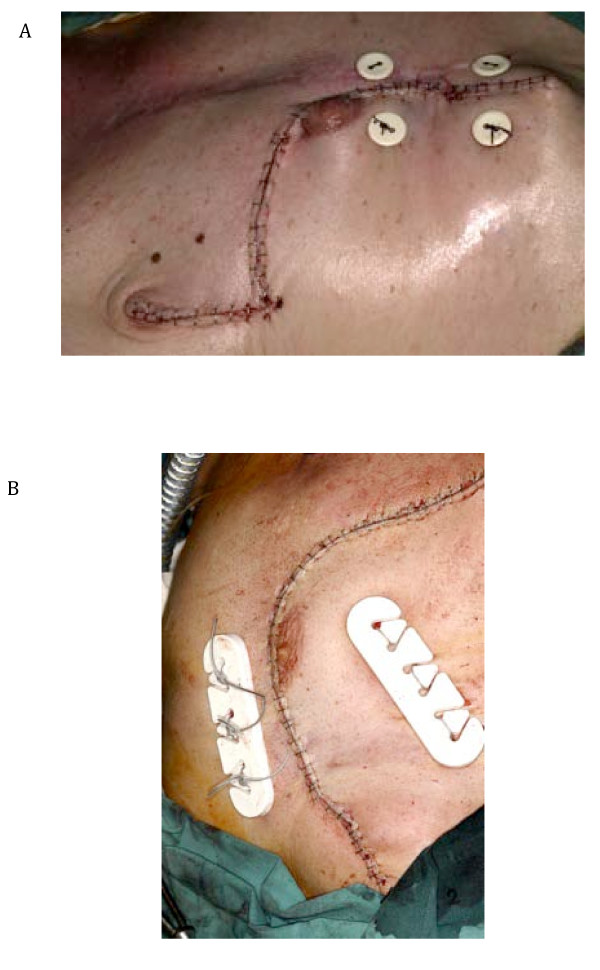
**a Closure of the donor side defect with buttons **b Closure of the donor side defect with Ventrofil^®^

Concerning closure of the donor-side, most authors performed a primary closure. But in some cases, different techniques have been described like buttons (Figure 8a) or Ventrofil^®^, a special tension-relief bridging device (Figure 8b) [27].

Several authors have described good results [28,29], but many have also mentioned high complication rates (Table 2).

**Table 2:** Overview of reported complication rates in PMMF

Authors	Year of publication	Number of patients/flaps	Reported complication rate
McLean et al. [9]	2010	136 patients 139 flaps	13%

Ethier et al. [5]	2009	27 patients	44.4%

Milenovic et al. [10]	2006	500 patients 506 flaps	33%

El-Marakby [4]	2006	25 patients 26 flaps	60%

Vartanian et al. [12]	2004	371 patients	36.1%

Dedivitis and Guimaraes [3]	2002	17 patients 17 flaps	41.2%

Liu et al. [8]	2001	229 patients 244 flaps	35%

Zbar et al. [13]	1997	21 patients 24 flaps	44%

Ijsselstein et al. [6]	1996	224 patients 224 flaps	53%

Kroll et al. [7]	1990	168 flaps	63%

Shah et al. [11]	1990	217 patients	53%

The current study supports that the harvesting technique is easy, but the postoperative complication possibilities as given in table 3 should not be underestimated [3].

**Table 3:** Known complications associated with pectoralis major myocutaneous flap

Problem	Suggested solution	References
*Partial necrosis*	Ties instead of electric cautery	Ord [17]

	Cutting muscle with Mayo scissors than electrosurgical knife	Carlson [28]

*Closure of donor-side*	Special attention to tension free closure	

*Supraclavicular bulge*	Excision of muscle over vascular pedicle	Wilson et al. [29]
	
	Turn flap under the clavicle	Wilson et al. [29]

*Female breast distorsion*	Only muscle flap	Phillips et al. [14]
	
	Inframammary approach	Zbar et al. [13]
	
	Lateral incision	Carlson [28]

Besides partial or complete necrosis, other complications such as fistula formation, dehiscence, infection, and hematoma are described [11,30]. The complication rate seems to be higher than in free flap reconstructions as, e.g., radial forearm flap [30].

Several reasons for complications have been described: while McLean et al [9] reported mainly complications in patients after radiotherapy, El-Marakby [4] mentioned utilization of the PMMF as a salvage procedure, number of comorbidities, oral cavity reconstructions. Zbar et al. found besides the mentioned reasons, complications mainly for covering exposed bone in osteoradionecoris [13].

A higher complication rate seems to be associated with the use of the flap as a salvage procedure and the presence of more than one risk factor - e.g. if the patient is a heavy smoker and or the procedure is oral cavity reconstruction [4] - while others reported no significantly higher complication rate associated with smoking, preoperative radiotherapy, or diabetes [8,12]. The incidence of flap necrosis is reported in up to 32% [11,31]. In the current study, in 6 patients out of 20 patients (30%), a complication appeared so that a further surgery was necessary. One explanation could be the variations in vascular supply as shown in Table 4.

**Table 4:** Blood supply of the pectoralis major according to Tobin [31] and Carlson [28]

Segment	Vascular supply	Nerve supply
Clavicular	Deltoid branch of thoracoacrominal artery	Lateral pectoral nerve

Sternocostal	Pectoral branch of thoracoacromial artery	Lateral pectoral and medial pectoral nerve

Lateral external	Lateral thoracic artery or/and pectoral branch of thoracoacrominal artery	Medial pectoral nerve

Therefore Ord recommended incorporating the lateral thoracic artery [19]. Furthermore, larger skin paddles introduce more perforators, and thereby possibly reducing the risk of necrosis.

Another reported point of concern is the problem of hidden recurrence under the flap [32].

Concerning the indication one must be aware on the one hand of the possible arc of rotation of the flap and, on the other hand, of the size of the defect. The latter has an approximate limit in men of 6 cm squared without the need of a further skin graft for closure: in females this size can be doubled due to greater redundancy of the female breast [33]. In regard to the possible arc of the rotation of the flap, soft tissue defects anterior to the retromolar region and inferior to the ear lobe and commissure of the lips can be reconstructed with relative ease [33].

Concerning the costs of PMMF in comparison to free flap, de Bree et al. have shown that the lower costs of hospital admission (24 days versus 28 days) in the postoperative phase outweighed the higher costs of the surgical procedure (692 min versus 642 min) in 40 radial forearm flap patients in comparison to 40 PMMF patients [34].

## Conclusion

The PMMF can be used in particular if a bone graft, a reconstruction plate for huge defects, or a bulky flap is needed for coverage of the carotid artery, but the complication rate should not be underestimated. In general, a microvascular free tissue transfer should be preferred.

Special attention should be given to the skin paddles in order to incorporate enough perforators. Extensive electrocoagulation should be avoided.

## Authors' contributions

AK carried out the evaluation of the patients, TL participated in the analysis of the tables, JO participated in the coordination, MB evaluated the surgical steps, and KG participated in the design and coordination of the study.

## Conflict of interest

The authors declare that there is no conflict of interest.
